# The impact of brief quality improvement (QI) projects by medical students in primary care in Gauteng or the North West Province, South Africa 

**DOI:** 10.4102/phcfm.v4i1.383

**Published:** 2012-08-03

**Authors:** Claire van Deventer, Nontsikelelo Sondzaba

**Affiliations:** 1 Family Physician, Dr Kenneth Kaunda District, North West Province, South Africa; 2 Department of Family Medicine, University of the Witwatersrand, South Africa; 3 Faculty of Health Sciences, University of the Witwatersrand, South Africa

## Abstract

**Background:**

The Integrated Primary Care (IPC) rotation is undertaken over six weeks by final year medical students at the University of Witwatersrand. Students are placed in either rural or urban primary health care centres based in Gauteng or the North West Province. As part of the IPC rotation, students undertake short quality improvement (QI) projects. The purpose of this study is to evaluate the impact of the QI projects undertaken over the period stretching from 2006 to 2010.

**Methods:**

An observational study of QI reports done by students. Project reports assessed and compared to site marks, indicators of learning assessed and individual and group marks compared.

**Results:**

Of 274 projects undertaken, 223 (81.4%) were available for evaluation. Geographical placements and QI themes were categorised. Management issues were most frequently identified as being problematic followed by chronic illnesses. Understanding and applying the principles of QI was partially achieved and gaps were identified for future projects. The most common intervention was training of personnel and design and distribution of posters or pamphlets.

**Conclusions:**

Most QI projects were well thought out and relevant to the chosen setting. In the majority of cases, a great deal of effort and creativity went into the process and skills other than clinical skills were employed such as writing, presentation of data in graphs and tables. Integration of theory and practice was achieved only partially.

## Abstrait

L’impact des courts projets d’amélioration de la qualité (AQ) par les étudiants en soins de santé primaires

**Présentation:**

La rotation des soins de santé primaire intégrés (SSP) se déroule sur six semaines par les étudiants en dernière année de médecine à l’Université du Witwatersrand. Les étudiants sont placés en centre de soins de santé primaires ruraux ou urbains dans la province du Gauteng ou du Nord-ouest. Dans le cadre de la rotation des SSP, les étudiants réalisent de courts projets d’amélioration de la qualité (AQ). L’objectif de cette étude est d’évaluer l’impact des projets d’AQ réalisés au cours de la période allant de 2006 à 2010.

**Méthode:**

Etude d’observation des rapports d’AQ réalisée par les étudiants. Les rapports de projet ont été évalués et comparés aux notes du site, aux indicateurs d’apprentissage évalués et les notes individuelles et de groupe ont été comparés.

**Résultats:**

Sur les 274 projets entrepris, 223 (81.4 %) ont pu être évalués. Les situations géographiques et les thèmes d’AQ ont été catégorisés. Les problèmes de gestion ont été les plus fréquemment identifiés comme étant les plus problématiques, suivis des maladies chroniques.  La compréhension et l’application des principes d’AQ ont été partiellement réalisées et des lacunes ont été identifiées pour les projets futurs. L’intervention la plus courante était la formation du personnel ainsi que la conception et la distribution d’affiches et de brochures.

**Conclusion:**

La plupart des projets d’AQ étaient bien pensés et pertinents quant à l’environnement choisi. Dans la majorité des cas, des efforts considérable et une grande créativité ont caractérisé ce processus, et des compétences autres que des compétences cliniques ont été utilisées, telles que les compétences rédactionnelles et de présentation des données sous forme de graphiques et de tableaux. L’intégration de la théorie et de la pratique n’a été que partielle.

## Introduction

### Background

The six week long Integrated Primary Care (IPC) rotation for final (sixth) year medical students at the University of the Witwatersrand is in its sixth year of implementation. About 30 students at a time are based as groups of 3 to 4 at a number of primary care sites in the urban Gauteng or rural North West provinces.

The objective of this rotation is to experience and practice integrated primary care that is responsive to patients and their families and communities. Students are expected to understand how to work with patients with undifferentiated problems across all medical disciplines, in settings where they would often be the first medical contact required to make crucial clinical management decisions.

The core of the training in this rotation is based on family medicine principles.^1^ Most of the clinical disciplines are covered by 30 different tasks set out in a logbook. One of these tasks is a short quality improvement (QI) project. Students audit the health facility in which they are based, within the first 2 weeks, by means of observation, interviews with key personnel, and review of existing information. Based on these findings students identify, in consultation with their local supervisor, either a new QI issue or one from the previous group’s project that they continue with, using the QI cycle.^2^

## Literature review

QI projects as learning experiences are based on sound educational principles. Learning, according to Varkey et al.^3^, is most easily accomplished when lessons can be placed in context and opportunities exist to apply the lessons learnt. Education of medical students in QI builds a culture of enquiry and innovation that is critical for the success of any health care organisation^4^. It is an extension of traditional clinical medicine into a broader awareness of systems issues and their resolution.

In the United States of America, medical educationists developed a curriculum for practice-based learning and QI for undergraduates, based on a review of 27 journal articles^4^. The outcome of this curriculum was that students were exposed to principles and theoretical teaching concerning QI from their first year, with gradual experiential exposure.

Weeks et al.^5^ demonstrated the outcomes of QI undertaken by students at a community practice site. They identified four factors that contribute to successful improvement of learning experiences for beginning medical students:

- didactic teaching concerning concepts and tools
- the availability of baseline data on patients 
- team cohesion and a sense of ownership
- resources and information for the improvement effort (e.g. literature, databases and administrative resources)

Most of the sites used for training were in hospitals, but there were successful interventions in rural areas as well.^6^

Another example of appropriate QI teaching is the Cleveland asthma project, in which each student in an eight week primary care block is required to describe a patient with asthma, investigate the cost of care, and assess the outcome by interviewing the patient. This helped develop a number of different skills, such as qualitative interviewing and costing, as elements of the final report.^7^

 An interesting study from two sites in Washington and Virginia demonstrated that students who receive adequate training can make important contributions to improvement teams, leading to positive patient perceptions.^8^

Knapp, Bennett, Plumb and Robinson^9^ found that certain factors that might have led to improved learning for students included ‘using health data to set project priorities, having a clear definition of a target community, selecting projects that can be completed in short periods of time that coincide with the structure of an academic year, and emphasizing interdisciplinary teamwork’.^9^ However, there were no data to demonstrate the effectiveness of specific teaching methods or learning outcomes. 

 In an article on residency training, the importance of recognising the ‘invisible staff’, namely the registrars, is emphasised in any improvement strategy.^10^ Many staff members are involved in projects by default, but their contributions are not formally recognised. ‘Invisible staff’ may also benefit by learning principles such as recognising standards of care, and evidence based practice. This is valuable for any student, either at under-graduate or post-graduate level, but recognition as part of a team is essential.

An initiative arising from a USA project to address undergraduate medical education for the 21st century  (UME 21), led to a major curriculum reform including the value of quality measurement and improvement as an innovative teaching tool if costs are considered.^11^ 

Kirkpatrick^12^ describes learner outcomes in a hierarchy of four levels, namely:

1. reaction (enjoyment and satisfaction)
2. learning (changed attitudes and knowledge) 
3. behavior change or performance (knowledge translated into practice)
4. systems change or results (improvement in patients’ health resulting from the actions of students).

The purpose of this study is to evaluate the educational impact of QI projects on final year medical students in primary care settings by means of a review of projects undertaken over the first five years of the IPC rotation (2006–2010). Particular reference will be made to the first two levels (reaction and learning) in Kirkpatrick’s hierarchy. The retrospective study design and data sources used preclude measuring individual behaviour change (level 3) and systems change (level 4).

## Methods

A review of the QI projects was undertaken by the researcher in order to describe the topics and the interventions undertaken, to assess the application of key QI principles, and to assess the grading of the projects by the site supervisors.

Key QI principles expected in the report were team involvement, standard setting, initial audit done and repeated, and a literature search. The methods of acquiring information, and the quality of the presentation of the report were also assessed.

The author, an experienced assessor who was blinded to the marks allocated by the site supervisors, graded each QI report using the marking schedule of the Family Medicine department. The marks of the researcher and site supervisor were then compared. Each student’s total mark for the block was also compared to his or her QI project mark in order to see if there would be a difference in group and individual learning. Differences were categorised as differing by less than 5%, between 5% and 9.9%, between 10% and 14.9%, and 15% or more.

## Results

A total of 274 projects were documented from 2006 to 2010,  of which 223 (81.4%) were evaluated, as the other 51 had been misfiled; 1047 students were involved in the IPC block over this time.

Gauteng Province had 149 urban placements, and North West Province 123 rural placements, at clinics and district hospitals or at outpatient departments. There were two placements at Tintswalo Hospital in Mpumalanga, a third province that was incorporated as part of the training complex in 2010.

### Categories of quality improvement

The range of topics reflects the broadness of the primary care system within which the students are working and gives an overview of untapped and important areas that may be suggested in future by site supervisors. 

The most common topic category chosen was management issues (Table 1). Topics in this category included queue management, drug supply, and referral systems. The second most popular category focused on chronic illnesses, such as diabetes and hypertension. Different types of flowcharts or patient booklets were designed by a number of the groups in order to create continuity of care for chronic patients.  Paediatric problems and HIV, AIDS, sexually transmitted illnesses, and/or tuberculosis (TB) (HAST) were relatively infrequently chosen. The paediatric projects mostly focused on improving the implementation of Integrated Management of Childhood Illness (IMCI). HAST projects included ‘cough hygiene’ education in the waiting room, improvement of record keeping regarding the TB register, information on sexually transmitted illnesses (STIs) to personnel, tracing interventions for partners of patients with STIs. Emergency care included the complete reorganizing of emergency rooms, including accessing of equipment and clinical guidelines as well as training. There were many projects under this banner that introduced or assessed triage systems in emergency departments.

**TABLE 1:**
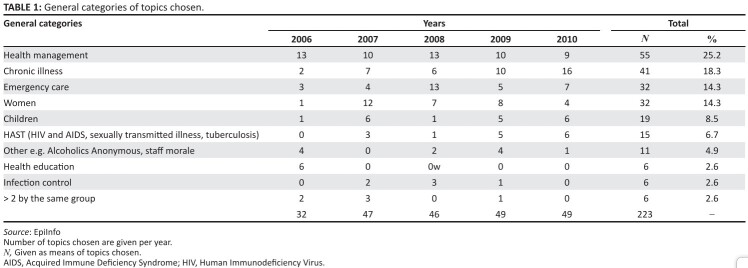
General categories of topics chosen.

Women’s issues commonly targeted the improvement of services relating to pap smears, use of the partogram, contraceptives, and perinatal issues, for example, prevention of mother to child transmission (PMTCT).

Health education, although part of many projects, was the primary focus in a small number of projects, where, for example, groups went to schools and interacted with large groups of pupils, especially regarding sexual health. Where groups attempted more than one project, there was less success in achieving their initial goals in terms of time and resources.

All but one of the reports had evidence of critical discussion regarding the previous group’s project and reflection on their own QI learning. Only 9.8% of projects were continuations of previous projects at the same site.

#### Quality improvement project interventions

The most common QI intervention was training (usually of personnel), and posters or pamphlets designed and distributed by the students. There was usually a combination of interventions, the above two most often done together.

#### Application of quality improvement principles in projects 

**FIGURE 1:**
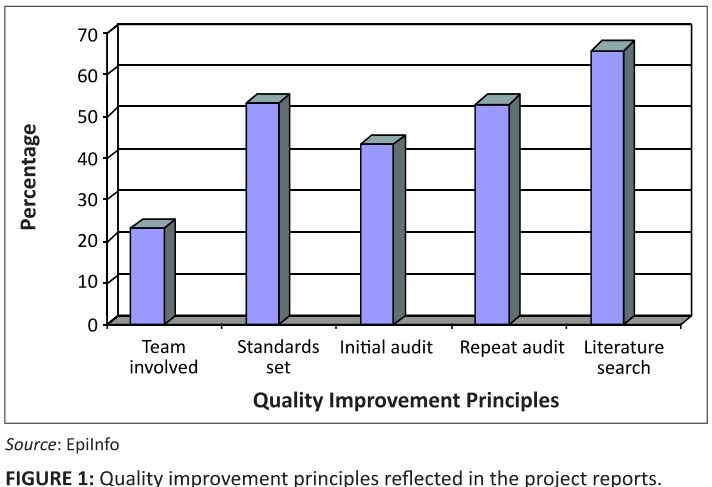
Quality improvement principles reflected in the project reports.

In terms of understanding and applying the principles of QI, only 23.3% of projects reflected inclusion of a broader health team in the QI, most being done primarily by the students themselves. Standards were set in 53% of the projects. Many others used research methodology rather than QI methodology and instead of standards and criteria, documented their aim and objectives. In all the projects a facility audit was done, as this was the trigger to identifying quality gaps. However, only 43% of the groups did a focused audit of the problem area that they had identified. The most common method for gathering information, apart from the facility audit, was by way of questionnaires to personnel or patients; 53% redid the initial audit, that is, completed one loop of a QI circle. Those that did not manage to do this, indicated that time had been their limitation. Literature was accessed or referred to with varying levels of competence by 63% of the groups (Figure 1).

Fifty-one point one per cent of the students documented their findings by means of graphs or tables in addition to a discussion of the findings. Addenda were added to support the projects in 79% of cases. The questionnaire used for the audit and/or pretest before training was the most common addendum. Other addenda were photos, posters or pamphlets that had been designed, minutes of meetings, clinical protocols and flowcharts.

#### Assessment of project scoring

The basic departmental marking schedule allows for a global mark of *‘Excellent’* (> 75%), *‘Good or Satisfactory’* (60% – 74.9%), *‘Borderline’* (50% – 59.9%), and *‘Fail’* (< 50%). Total scores differed widely on occasion between the researcher and the site supervisor, with the sites generally giving higher marks (Table 2). Most of the site assessments were in the ‘Excellent’ range, whereas the researcher had a more even distribution, with slightly more projects falling in the *‘Good or Satisfactory’* bracket; 27% of the marks differed by more than 10%. There were five sites that had the greatest discrepancy, although this was not constant over all five years. 

**TABLE 2:**
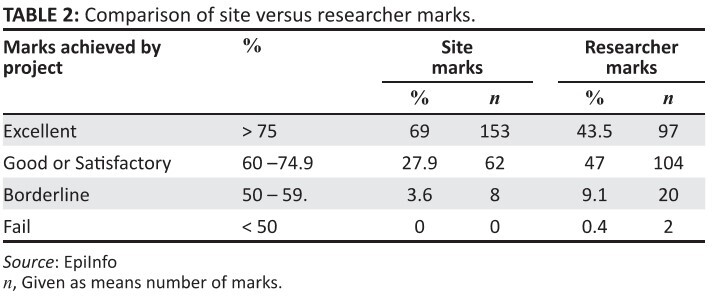
Comparison of site versus researcher marks.

Although these findings were not statistically significant, the comparative total mark for each student for the block compared to their QI group project mark found that across the five years, there was a statistically significant higher mark for the QI project than for the individual total mark for the block (*p* = 0.0001)(Table 3). It was found that the group mark differed at times as much as 20% from the individual’s  total mark.

**TABLE 3:**
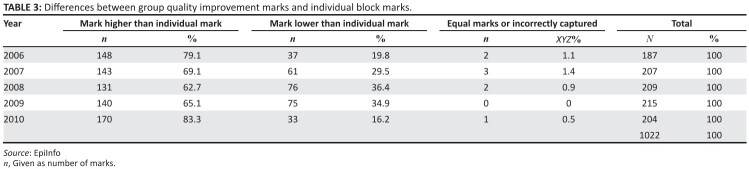
Differences between group quality improvement marks and individual block marks.

## Discussion

All the QI projects were a response to some weakness that the students had identified in their particular system, although not all of them had come directly from the facility audit. The process they all followed was one of identifying a problem, having a clearly designated team with specific team tasks (sometimes actively involving the staff at the clinics), setting standards, doing some kind of audit to assess the current situation and compare it to the standards set, making a plan to correct the discrepancies, and then evaluating the success of the plan.^2^

Chronic illness was a popular choice but chronic obstructive airways disease, cardiac disease and epilepsy were not included in this. HIV and AIDS, sexually transmitted illness and TB (HAST) and childcare were relatively poorly represented as topics. Areas like mental health and forensic medicine, which are relevant at primary care level, were not represented at all. Occasionally one group attempted to address more than one issue. The fact that the most popular topic was management issues may be a reflection on the system in which students find themselves. Nevertheless, this is an excellent introduction to the non-clinical role of a primary care doctor which may help future doctors to make an impact on health systems. Factors that may have influenced the topic choices could be the most recent rotation, individual interests and skills as in the extensive electronic filing system developed at one clinic, and others. The site supervisor is generally requested to support student’s choices irrespective of how they originate, if they are viable.

Evidence of learning, reflecting level 2 of Kirkpatrick’s hierarchy, was present in the reports written by students, as the principles of QI were applied in all the studies to a lesser or greater degree. The presentations of projects were varied, with some of them incorporating posters and training material as well as graphs indicating improvement in knowledge or practice. Others included photographs of their activities. Those that were very basic with superficial information and no attachments or pictures were in the minority. However, most of the reports reflected a great deal of effort and pride which would imply an impact on the first level in Kirkpatrick’s hierarchy. Group dynamics influenced the overall success of the project as did the topic. Groups where one of the students had professional experience on a chosen topic, such as IT systems, had the most success in the engagement and presentation of their project.

A difficult shift and a common problem in medicine is the involvement of the team. This was reflected in most projects where facility personnel were superficially involved. At a few sites the local family physician requested particular areas to be assessed and improved, so in such instances the students did not have a choice of topic. This need not detract from the QI process as a whole, but it does remove one of the key aspects, namely appropriate identification of priority problems. 

 Understanding the setting of standards and the use of criteria in a QI process will need some attention as only 53% of students did this correctly. The difference between research methodology and QI was a problem in some cases. Possibly as a result of this, only 43% of students did a dedicated audit of their chosen topic; the others used the facility audit as their baseline which was not always appropriate. 

The innovation of certain groups was exemplary; an Alcoholics Anonymous (AA) support group was set up by one group and strengthened by a second group; an entire electronic filing programme for a clinic was created and implemented within a few weeks; and a motivational tea party was given to help staff morale. In some facilities structural improvements included painting, putting up shelves and partitions to improve work flows and waiting times, and organising medical and drug stores and wards; all of this was undertaken at the students’ expense. Many resources have been created in the form of protocols, flow sheets, handouts for patients, and teaching aids for staff.

The presentation of the results was done using graphs and tables in about half the projects, which is an additional non-clinical skill that many doctors require in their careers. In terms of outcomes, added value was found at the few sites where a previous QI had been repeated, as the continuity strengthened the overall outcome. This may deprive the students of assessing and managing a problem at the start of the project, but seems to have a positive effect on the sustainability at the site. Where there had been structural changes such as the re-organisation of an emergency room or pharmacy, there was more sustainability than where pre- and post-test teaching was done. This was an unexpected finding which was not provided for in the design of the study and was suggestive of a Kirkpatrick level 4 activity.

### Lessons learned

Short QI projects are possible. Medical students are usually not keen to continue a project started by another group as it has been the author’s experience that they enjoy newly discovered challenges. It is possible to evaluate a group’s learning on many levels by means of this process. In particular, the first two levels of the Kirkpatrick hierarchy are well achieved, and there is some suggestion of systems change to the advantage of patients and staff (level 4). As the study was not assessing levels 3 and 4, these may be areas for future investigation.

With the large discrepancy between group and individual marks, there may have been students who participated less and therefore benefited less. Individual projects would allow more accurate assessment of individual learning (Table 3). 

‘Facility fatigue’ may become a threat as many reports reflected students’ inflated perception of the extreme gratitude of staff for what the students were accomplishing. Personnel have been very accommodating in spite of what appears to be an energetic youthful arrogance evident in a number of reports. However, sensitive circulation, including rest periods for sites, would be advisable over the long term.

Realism has on occasion interfered with projects, as in the case of a group intending to publish weekly health reports in a local paper without understanding the processes and politics of journalism. They did, however, manage to publish a few short health reports over two projects.

In evaluating the QI projects, it is valuable to place the primary emphasis on the learning experience of students regarding quality projects in primary care and have as a secondary emphasis the advantages this process has for the clinic, patients and staff.8 What is evident is that as long as the focus is on QI projects and not on QI programmes, which are unfortunately determined by the structure of the system, sustainability is not guaranteed and some of the initiatives may therefore dissipate.^13^

### Practical implications

With the information gained, the orientation and supervision of students can be focused on areas of misunderstanding or weakness, that is, team involvement, setting of standards, avoidance of unrealistic planning and valuable interventions that may be sustainable.

### Limitations of the study

Given that the time of the QI projects was never longer than 6 weeks and often as short as 3–4 weeks, the QI projects were not always completed. The following group at a site was encouraged to complete a previous group’s partial QI project, but few of them did so.

The discrepancy in marks indicates that some assistance needs to be given to supervisors at sites. There is difficulty in getting to know an enthusiastic group of students and then having to mark their work objectively if the QI project was not well documented, as personal feelings interfere with this objectivity.

### Recommendations

The report is to be discussed with all the involved supervisors in the department, and at the sites, in order to identify the weaknesses reflected by the students. Where supervisory issues have been pinpointed at sites, these will be directly addressed at those points.

An impact study at the sites in terms of either the sustained positive influence of the quality improvement projects, or the transitory nature of these changes, is strongly considered as a future research project. 

## Conclusion

Nearly all of the QI projects seemed to be well thought out and relevant to the chosen setting. Short term benefits were realised by both the staff and patients. Management teams expressed appreciation for the inputs. Students had real life challenges in health care. There was a perceived mutual benefit with both the students and the services benefiting although the quantification thereof would be a separate study. The student groups became temporary change agents. The educational strategy has been to expose students to the theory of QI by involving them in clinical practice. When the QI reports are studied, this has generally been well grasped and implemented and where deficits have been identified through this study, an effort can be made to better guide and supervise the following groups.

It was interesting to see that there were projects that worked well and that were partly sustained and others not at all. The factors that lead to successful versus less successful outcomes need to be understood; this is a topic for another study.
